# Myelodysplastic Syndrome-Associated SRSF2 Mutations Cause Splicing Changes by Altering Binding Motif Sequences

**DOI:** 10.3389/fgene.2019.00338

**Published:** 2019-04-16

**Authors:** So Masaki, Shun Ikeda, Asuka Hata, Yusuke Shiozawa, Ayana Kon, Seishi Ogawa, Kenji Suzuki, Fumihiko Hakuno, Shin-Ichiro Takahashi, Naoyuki Kataoka

**Affiliations:** ^1^Laboratory for Malignancy Control Research, Medical Innovation Center, Kyoto University Graduate School of Medicine, Kyoto, Japan; ^2^Laboratory of Molecular Medicinal Science, Department of Pharmaceutical Sciences, Ritsumeikan University, Shiga, Japan; ^3^Department of Pathology and Tumor Biology, Kyoto University Graduate School of Medicine, Kyoto, Japan; ^4^Laboratory of Cell Regulation, Departments of Applied Animal Sciences and Applied Biological Chemistry, Graduate School of Agriculture and Life Sciences, The University of Tokyo, Tokyo, Japan

**Keywords:** myelodysplastic syndrome, splicing, SRSF2, exonic splicing enhancer, aberrant splicing, EZH2 (enhancer of zeste homolog 2)

## Abstract

Serine/arginine-rich splicing factor 2 (SRSF2) is a member of the SR protein family that is involved in both constitutive and alternative mRNA splicing. Mutations in SRSF2 gene are frequently reported in myelodysplastic syndromes (MDS) and acute myeloid leukemia (AML). It is imperative to understand how these mutations affect SRSF2-mediated splicing and cause MDS. In this study, we characterized MDS-associated SRSF2 mutants (P95H, P95L, and P95R). We found that those mutants and wild-type SRSF2 proteins showed nuclear localization in HeLa cells. *In vitro* splicing reaction also revealed that mutant proteins associated with both precursor and spliced mRNAs, suggesting that the mutants directly participate in splicing. We established the human myeloid leukemia K562 cell lines that stably expressed myc-tagged wild-type or mutant SRSF2 proteins, and then performed RNA-sequence to analyze the splicing pattern of each cell line. The results revealed that both wild-type and mutants affected splicing of approximately 3,000 genes. Although splice site sequences adjacent to the affected exons showed no significant difference compared to the total exons, exonic motif analyses with both inclusion- and exclusion-enhanced exons demonstrated that wild-type and mutants have different binding sequences in exons. These results indicate that mutations of SRSF2 in MDS change binding properties of SRSF2 to exonic motifs and this causes aberrant splicing.

## Introduction

Serine/Arginine rich (SR) proteins are essential splicing factors that also confer regulatory activity of alternative splicing (Manley and Krainer, 2010; Howard and Sanford, 2015; Kataoka, 2017). SR protein family consists of 11 proteins in human. SR proteins contain one or two RNA binding domain (RBD) at amino-terminus and multiple repeats of Serine-Arginine dipeptides at carboxy-terminus. SRSF2, which was originally called SC35, is a member of the SR protein family (Fu and Maniatis, 1990; Fu and Ares, 2014; Kataoka, 2017). During splicing, SRSF2 promotes exon recognition by binding to exonic splicing enhancer (ESE) motifs in precursor of mRNA (pre-mRNA) through its RBD. This promotes both the binding of U2AF heterodimer and U1 snRNP to the upstream 3′ splice site and to the downstream 5′ splice site, respectively (Chen and Manley, 2009; Fu and Ares, 2014; Kataoka, 2017).

Recently SRSF2 was found to be one of the major responsible genes of myelodysplastic syndrome (MDS). MDS is a heterogeneous group of chronic myeloid neoplasms characterized by many symptoms, such as ineffective hematopoiesis, peripheral blood cytopenia and a high risk of progression to acute myeloid leukemia (Fu and Maniatis, 1990; Cazzola et al., 2013). Mutations in splicing factors represent a novel class of driver mutations in human cancers and affect about 50% of patients with myelodysplasia (Graubert et al., 2011; Papaemmanuil et al., 2011, 2013; Yoshida et al., 2011; Walter et al., 2013; Haferlach et al., 2014). Somatic mutations are frequently found in genes SF3B1, SRSF2, U2AF1 and ZRSR2 (Graubert et al., 2011; Papaemmanuil et al., 2011; Yoshida et al., 2011). Interestingly, the common feature of those gene products in pre-mRNA splicing is a 3′ splice site recognition, implicating that MDS pathogenesis is caused most likely by abnormal pre-mRNA splicing. The mutations are heterozygous and clustered in specific amino acid residues for SF3B1, SRSF2 and U2AF1 (Yoshida et al., 2011; Darman et al., 2015; Ilagan et al., 2015; Kim et al., 2015). These ‘hotspot’ mutations are predicted to be a ‘gain-of-function’ mutations by affecting protein structure (Yoshida et al., 2011; Darman et al., 2015; Ilagan et al., 2015; Kim et al., 2015). Among them, the hotspot mutations in SRSF2 were found in proline residue residing slightly outside of its RNA binding domain. Changes in alternative splicing patterns with SRSF2 mutations were reported in culture cells, mouse models and primary human samples (Przychodzen et al., 2013; Brooks et al., 2014; Shao et al., 2014; Ilagan et al., 2015; Kim et al., 2015; Komeno et al., 2015; Shirai et al., 2015; Zhang et al., 2015; Obeng et al., 2016; Mupo et al., 2017). In spite of these important findings, the precise mechanism for aberrant splicing in the cells carrying MDS mutations still remains largely unclear.

To address the mechanism of aberrant splicing in MDS, we have prepared expression plasmids for both wild-type and MDS-causing mutants of SRSF2, P95H, P95L and P95R. Transcriptome analyses using total RNAs recovered from those cell lines revealed that both wild-type and mutants affected splicing of approximately 3,000 genes. Motif analyses with both inclusion- and exclusion-enhanced exons demonstrated that the mutants have different binding sequences in exons compared to wild-type protein. These results strongly suggest that MDS mutations in SRSF2 alter binding properties of SRSF2 to exonic motifs and this results in aberrant splicing.

## Results

### Localization of Wild-Type and Mutant SRSF2 Proteins in HeLa Cells and Association With Pre-mRNA and mRNA *in vitro*

**FIGURE 1:**
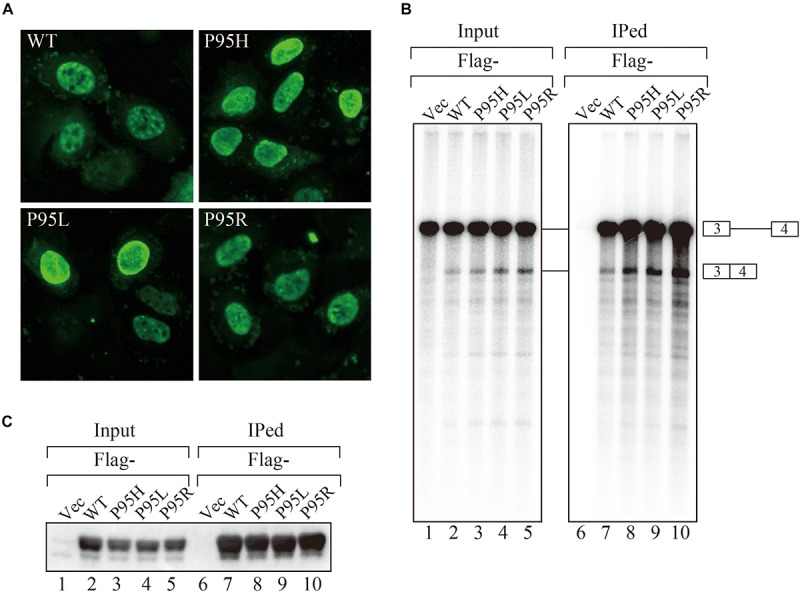
MDS-causing SRSF2 mutant proteins are localized in the nuclei and associate with pre-mRNA and mRNA *in vitro*. **(A)** HeLa cells were transfected with expression vectors encoding either wild-type or mutant SRSF2 proteins (P95H, P95L, and P95R) fused with myc-tag. **(B)** *In vitro* splicing reaction in the presence of either Flag-tagged wild-type SRSF2 protein or mutant proteins followed by immunoprecipitation of RNAs by anti-Flag M2 antibody. **(C)** Immunoprecipitation of SRSF2 proteins by anti-Flag antibody from in vitro splicing reaction. Five percent of total reaction mixture was used as an input. The anti-Flag M2 antibody was used for detection of Flag-tagged SRSF2 proteins.

In order to gain insights on how mutations in SRSF2, one of the splicing factors mutated in MDS patients, affect splicing, we have prepared the SRSF2 mutant cDNAs carrying three kinds of mutations at 95th position of Proline residue (P95H, L and R) found in MDS patients. We first determined their subcellular localization in HeLa cells. Both wild-type and mutants of SRSF2 cDNAs were transfected into HeLa cells and those proteins were expressed as fusions with a myc-tag. When the myc-SRSF2 wild-type protein was expressed, it was localized in both speckles and nucleoplasm, likely due to overexpression (Figure 1A, panel a). The subcellular localization of mutant SRSF2 proteins was similar to that of the wild-type, although mutant proteins exhibited slightly less numbers of nuclear speckles (Figure 1A).

We have also carried out *in vitro* splicing reaction with both Flag-tagged wild-type and mutant SRSF2 proteins, followed by immunoprecipitation of RNA from the mixture in order to test whether mutant proteins were able to support splicing or not. For this assay, we used the immunoglobulin μ chain (IgM) pre-mRNA, whose splicing is SRSF2-dependent (Mayeda et al., 1999). HeLa cell extract was mixed with cell lysates from HEK293T cells that express either wild-type or each mutant SRSF2 protein. The Flag-vector transfected cell lysate was used as a negative control. The results of splicing reaction are shown in Figure 1B. With a negative control, splicing did not take place (lane 1), indicating the dependency of IgM pre-mRNA to SRSF2. As shown in Figure 1B (lanes 2–5), both wild-type and each mutant supported IgM splicing *in vitro*, indicating mutant SRSF2 proteins have a splicing supporting activity even stronger than wild-type protein. Immunoprecipitation was carried out with the reaction mixtures using anti-Flag tag antibody. Flag-SRSF2 wild-type precipitated both pre-mRNA and mRNA (Figure 1B, lane 7). Mutant SRFS2 proteins also precipitated pre-mRNA and mRNA more efficiently than wild-type protein (Figure 1B, lane 8–10). Immunoprecipitated SRSF2 proteins were also detected by western blotting with anti-Flag M2 antibody (Figure 1C), and it turned out the comparable amounts of both wild-type and mutant SRSF2 proteins were precipitated (Figure 1C, lanes 7–10). Take all the results together, we concluded that mutant SRSF2 proteins are able to cause change of splicing pattern in cells, and the mutants likely have higher affinity to SRSF2-mediated ESE sequences.

### Detection of Splicing Pattern Changes in K562 Cells Expressing Mutant SRSF2 Proteins

**FIGURE 2:**
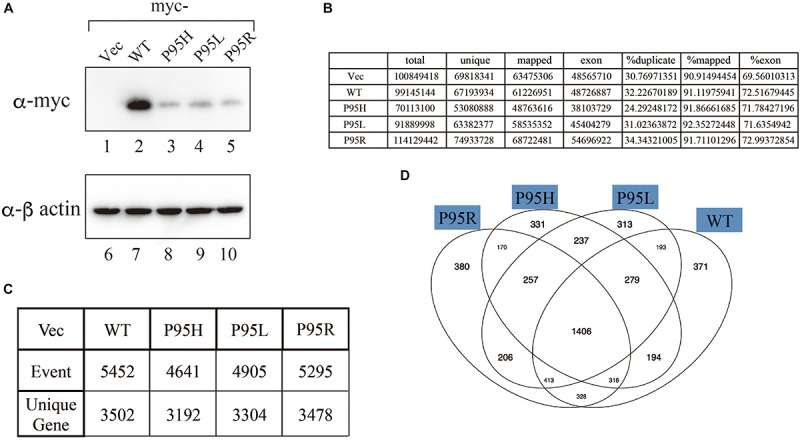
Analyses of splicing changes in K562 cells stably expressing either wild-type or mutant SRSF2 protein. **(A)** Immunoblotting analyses of K562 cell lines stably expressing myc-tagged wild-type or mutant SRSF2 proteins. A myc-vector was used as a negative control. **(B)** Numbers of reads from RNA sequence analyses and mapping percentage of sequence tags. **(C)** Numbers of altered splicing events and genes identified by comparing with myc-vector transfected cells. **(D)** Venn diagram comparing the differentially spliced genes among myc-vector, WT and three SRSF2 mutants expressing cells.

We generated to generate cell lines that stably express either wild-type or SRSF2 mutants with K562 cells, a myelogeneous leukemia cell line. After establishment, we checked the protein expression level by western blotting by using anti-myc tag antibody. As shown in Figure 2A, all cells expressed myc-tagged SRSF2 proteins except myc-vector transfected cells. During selection of the stable cell lines, cells expressing relatively large amount of mutant SRSF2 proteins tend to die, likely due to toxicity of mutant proteins and cells expressing high amount of mutant proteins may have been eliminated during selection of the clones. Total RNAs derived from those cell lines were applied to RNA sequence analyses. The reads were mapped to human genome with more than 90% efficiency (Figure 2B). We determined splicing changes by comparing wild-type and mutants splicing patterns with vector transfected one. It turned out that more than 5000 splicing events in more than 3000 genes were detected with both wild-type and mutants (Figure 2C). Drawing Venn diagram demonstrated that overlapping genes for all of them are 1406 genes, and there are also many genes either specific to each protein (Figure 2D).

### ESE-Like Motifs Were Enriched in Exons Skipped by SFSF2 Mutant Proteins

**FIGURE 3:**
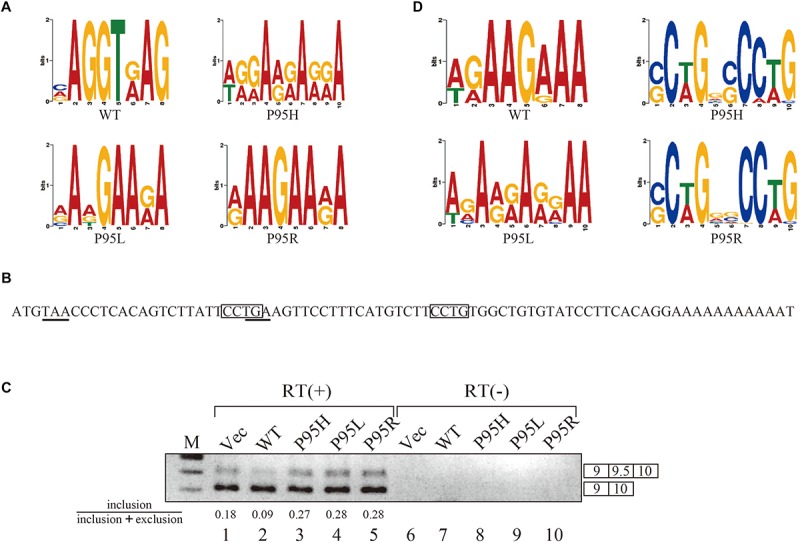
SRSF2 mutants promote exon 9.5 of EZH2 gene through binding to CCWG motif. **(A)** Sequence logos for the motifs enriched in inclusion-type exons with WT, P95H, P95L, and P95R are presented. **(B)** Sequence logos for the motifs enriched in inclusion-type exons with WT, P95H, P95L, and P95R are shown. **(C)** Sequence of exon 9.5 (pseudo exon) in human EZH2 gene. In frame stop codons are underlined. CCWG motif, found with SRSF2 P95H and P95R proteins in **(B)**, are indicated with open boxes. **(D)** Splicing pattern of EZH2 pre-mRNA in myc-vector, SRSF2 WT, P95H, P95L, and P95R are indicated. The ratio of exon9.5 included product in total splicing products is shown beneath the panel. The schematic representations of PCR products are shown at the right side of the panel. RT, Reverse Transcriptase. M, DNA size marker.

Since we identified many genes whose splicing patterns were affected by the expression of both wild-type and mutant SRSF2 proteins, we investigated the motifs of exons enriched in both wild-type and mutants regulated genes. Specific sequence features in the exon were searched by multiple expectation-maximization for motif elicitation (MEME) (Bailey et al., 2006) algorithm to find the enrichment or depletion of novel sequence motifs. Surprisingly, enriched motifs for all mutants are purine-rich sequences (Figure 3A). The motif for wild-type SRSF2-excluded exons is AGGTRAG (R indicates purine residue), in which the purine stretch is separated by T residue (Figure 3A). It has been shown that SRSF2 proteins are able to bind purine-rich ESEs (Cavaloc et al., 1999). These results strongly suggested that MDS-causing mutations in proline residue of SRSF2 cause reduction of the affinity to purine-rich ESEs.

### Mutant SRSF2 Proteins Prefer CCWG Motif for Exon Inclusion

We also carried out motif analysis for included exons, and we found that a purine-rich motif appeared as the most frequently appeared motif with a wild-type protein (Figure 3B), consistent with the previous findings that SRSF2 binds to purine-rich ESE to promote exon inclusion (Cavaloc et al., 1999). With SRSF2 P95L, a similar A/G rich motif was also found (Figure 3B), suggesting this mutation has slight effect on recognizing sequence of SRSF2 protein. In contrast, with two other mutant proteins, SRSF2 P95H and P95R, CCWG (W: weak as T or A) containing motif was identified in inclusion-promoted exon at the top (Figure 3B). These results strongly suggested that purine-rich motif and CCWG-containing motif function as ESE for wild-type and mutants, respectively, and MDS-causing mutations alter the high affinity of SRSF2 from purine-rich motif to CCWG motif.

We have confirmed the splicing change with wild-type and mutant proteins by choosing several genes to determine splicing changes by RT-PCR. Among them, the splicing change of EZH2 gene is shown in Figure 3D. EZH2 is also known as one of the responsible genes for causing MDS (Ernst et al., 2010). With RNA sequencing analysis, the number of reads for exon 9.5, which has two CCWG motifs and premature termination codons (Figure 3C), was reduced with SRSF2 wild-type expression. In contrast, mutant protein expression increased the numbers of exon 9.5 reads, suggesting that mutant proteins promote exon 9.5 inclusion whereas wild-type enhances skipping of this exon. To test this possibility, we carried out RT-PCR analysis by amplifying Exon9–Exon10 region of EZH2 mRNA. The results indicate that wild-type protein expression reduced the ratio of exon 9.5 included mRNA (Figure 3D, lane 2). In contrast, all of the mutant proteins increased exon 9.5-included mRNA ratio (Figure 3D, lanes 3–5). These results indicate that SRSF2 wild-type and mutants have an opposite effect on CCWG-containing exon splicing.

## Discussion

**FIGURE 4:**
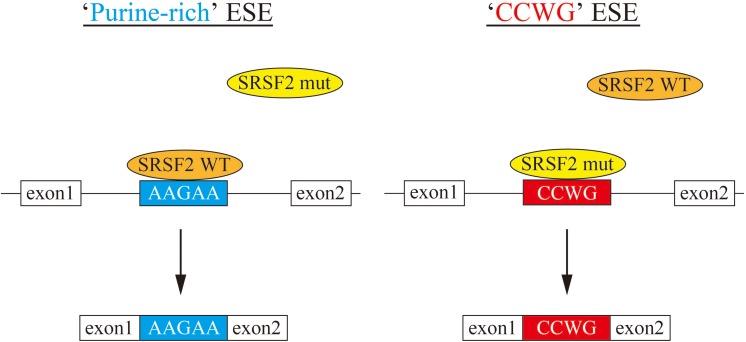
A model for splicing changes caused by SRSF2 mutations in the patients. **(Left)** SRSF2 wild-type protein binds to purine-rich exonic splicing enhancer for promotion of exon recognition. **(Right)** SRSF2 mutants, SRSF2 P95H and P95R, preferentially bind to CCWG motif-containing exons to promote aberrant splicing.

In this manuscript we have analyzed the splicing changes in K562 cells stably expressing either wild-type or mutant SRSF2 proteins. As expected, both wild-type and mutants affected many splicing events of various genes, and CCWG motif was found in inclusion promoted exons with mutant proteins. CCNG or GGNG motif was previously reported as the binding sequences of SRSF2 protein by SELEX (Cavaloc et al., 1999). Indeed, three mutant proteins promote CCWG-motif containing pseudo exon of EZH2, which is one of the responsible genes for MDS (Figure 3D) (Ernst et al., 2010). This mis-splicing may affect EZH2 protein level in cells and the reduction of EZH2 protein level would results in mis-regulation of epigenetics (Kim et al., 2015; Shirahata-Adachi et al., 2017; Kon et al., 2018; Shiozawa et al., 2018). All three SRSF2 mutants were able to support SRSF2-dependent substrate (IgM) splicing *in vitro* more efficiently and associate with IgM pre-mRNA and mRNA more strongly than wild-type protein (Figure 1B). There are several CCWG motifs in 3′ exon of IgM pre-mRNA (Mayeda et al., 1999). It is highly likely mutant proteins bind to these motifs more strongly than wild-type (Figure 4). On the other hand, the AGGTRAG motif was identified in exons excluded by wild-type SRSF2 (Figure 3A). In this motif, purine-stretch is separated by T (U in RNA). It was demonstrated that U residue splitting purine-stretch in ESE abolishes splicing-promoting activity *in vitro* (Watakabe et al., 1989). This motif may bind to SRSF2 with low affinity. Alternatively, it was demonstrated that SR protein overexpression can cause exon skipping which depends on their prevalent actions on a flanking constitutive exon, and it requires collaboration of more than one SR protein (Han et al., 2011). The AGGTRAG sequence might be a binding motif of ‘weak’ SR protein, not a direct binding motif for SRSF2 protein, to support exon inclusion. When overexpressed, wild-type SRSF2 protein can bind to the flanking exon as a ‘strong’ SR protein more efficiently. The purine-rich ESE like sequence motifs were identified in exclusion promoted exons with mutant proteins (Figure 3A). Mutant proteins may have lower affinity to purine-rich sequences than wild-type (Figure 4). It is assumed that MDS mutations in Proline residue affect the conformation of RNA binding domain of SRSF2, although this proline residue is outside of RNA binding domain consensus. Indeed, 3D clustering analysis with protein structure predicted that this Proline residue is able to contact with RNA (Kamburov et al., 2015). Therefore, this residue is a part of RNA binding domain. By changing Proline residue to Histidine, Arginine or Leucine, the affinity with specific RNA sequence can be altered. Comparing crystal structures of these mutants-RNA complexes with that of wild-type would reveal the mechanism for the recognition of different RNA sequences. We believe this also uncover why mutant SRSF2 proteins have lower affinity to purine-rich ESEs.

Despite of the common features described above, each mutant contains peculiar subset of genes whose splicing patterns were specifically changed (Figure 2D). These splicing changes in certain genes may confer the pathological difference among the MDS patients. Further analyses of specific splicing changes in each mutant are also required.

Most recently several groups demonstrated that MDS responsible mutations in SRSF2 and U2AF1 cause augment of R loop (Chen et al., 2018; Nguyen et al., 2018). Enhanced R loop formation activate the ataxia telangiectasia and Rad3-related protein (ATR)-Chk1 pathway, which likely contributes to MDS phenotype (Nguyen et al., 2018). Efficient formation of R loop can occur by slowing down rearrangement of mRNA-protein complexes during/after splicing. The different binding affinity of SRSF2 mutants to RNA may be involved in this step. It would be of a great interest in which portions of genes form R loops and whether enrichment of CCWG motif can be observed in those regions or not.

## Materials and Methods

### Plasmid Construction

The cDNA of human SRSF2/SC35 was amplified by Reverse Transcription and Polymerase Chain Reaction (RT-PCR). The cDNA was cloned between BamHI and XhoI sites of either myc- or Flag-pCDNA3 vector. The resultant plasmid was used as a template in order to prepare mutant cDNAs that harbor MDS mutations, such as P95H, P95L and P95R. Point Mutations were introduced by QuikChangeTM Site-Directed Mutagenesis Kit (STRATAGENE) with myc-SRSF2 plasmid in accordance with the manufacturer’s recommendations.

### Cell Culture and Establishment of Stable Cell Lines

K562 cells were cultured at 37°C with 5% CO_2_ in RPMI1640 supplemented with 10% (v/v) fetal bovine serum (Sigma-Aldrich) and 1% (v/v) penicillin/streptomycin antibiotics (standard medium). K562 cells were transfected with pcDNA3-myc-SRSF2(WT), pcDNA3-myc-SRSF2(P95H), pcDNA3-myc-SRSF2(P95L), pcDNA3-myc-SRSF2(P95R) or empty vector plasmid, using Lipofectamine 2000 (Invitrogen) according to the manufacturer’s instruction. Transfected K562 cells were selected with 100 mg/mL G418, and then resistant cells were isolated with limiting dilution methods in 96-well plates. Obtained each stable clone-cells were maintained in 50 mg/mL G418 RPMI medium.

For HEK293T cells, DMEM supplemented with 10% (v/v) fetal bovine serum (Sigma-Aldrich) and 1% (v/v) Antibiotics Antimycotics (SIGMA) was used for cell culture and Lipofectamine 2000 was also used for transfection. Total cell lysates from transfected HEK293T cells were prepared as previously described (Kataoka and Dreyfuss, 2004, 2008; Kataoka, 2016).

### *In vitro* Transcription, Splicing and Immunoprecipitation of RNAs

For *in vitro* transcription, pμC3-C4 plasmid linearized by HindIII was used as a template and performed as described previously (Watakabe et al., 1989, 1993). *In vitro* splicing reaction with HeLa cell nuclear extracts complemented with Flag-SRSF2 protein expressing HEK293T total cell lysates was carried out as previously described (Kataoka et al., 2001, 2011; Kim et al., 2001; Kataoka and Dreyfuss, 2004; Kawano et al., 2004; Kataoka, 2016). Immunoprecipitation of RNAs from splicing reaction was accomplished by using anti-Flag M2 agarose beads (SIGMA) by the protocol described previously (Kataoka et al., 2001, 2011; Kataoka and Dreyfuss, 2004; Kataoka, 2016).

### Antibodies, Western Blotting and Immunostaining of HeLa Cells

The antibodies used for immunoblotting and immunostaining are as follows: anti-myc (MC045, Nacalai Tesque, Japan), anti-Flag M2 (Sigma-Aldrich), fluorescein isothiocyanate-conjugated goat anti–mouse F(ab’)^2^ (Cappel Laboratories, Durham, NC, United States), peroxidase-conjugated goat anti–mouse IgG antibodies (Jackson Immuno Research Laboratories, West Grove, PA, United States). For western blotting, the cells were lysed in CelLytic M Cell Lysis Reagent (Sigma-Aldrich) containing a protease inhibitor cocktail (Roche). The lysates were boiled with SDS-sample buffer at 95°C for 3 min. The samples were subjected to SDS-PAGE, transferred to PVDF membranes by iBlot system (Invitrogen), and incubated with primary antibodies. The membranes were washed and incubated with horseradish peroxidase-conjugated secondary antibody. Finally, chemiluminescence was detected using Chemi-Lumi One Super kit (Nacalai Tesque), and luminescence images were analyzed by LAS 4000 (GE Lifesciences). Immunostaining of HeLa cells with anti-myc antibody was performed as described previously (Kataoka et al., 2011).

### RNA Recovery, RNA Sequence and Alternative Splicing Analysis

Total RNAs from K562 stable cells were performed by using RNeasy Mini Kit (QIAGEN). The synthesis and amplification of complementary DNA were performed using SMARTer Ultra Low Input RNA Kit for Sequencing, version 3 (Clontech). Each sample applied illumina GA. The reads were trimmed to 99 bases and were mapped on hg19 genomes and gencode v7 protein coding transcripts by tophat (v2.0.9). To computate junctions in each sample, we processed the gencode GTF file by eval package (v2.2.8) and applied the mapped reads to juncBASE (v0.6) packages by following the options: ‘–min overhang = 6 -l 99 -c 3.’ For comparing each sample, we calculated “Percent Splicing index” (PSI) and corrected *p*-value and Benjumin & Hedgehog multiple test using pairwise fishers test getASEvents w reference.py by following options: ‘–jcn seq len = 186 –method = BH’ comparing with vector and wild-type.

### Motif Analysis

To search RNA binding motif of mutated SRSF2, we collected 100 base exon side sequences of exclusion junctions separating upper or lower than vector’s PSI in each sample and the upper and lower sequences was applied MEME v4.10.0 as following that options: ‘-*minw* 4-*maxw* 10 -*maxsize* 1000000.’

### Reverse Transcription Polymerase Chain Reaction (RT-PCR)

RT-PCR reaction was accomplished as described previously (Wang et al., 2017). Briefly, 1 μg of total RNA was used for reverse transcription with prime Script Reverse Transcriptase (TAKARA, Japan). The produced cDNA was used for PCR reaction by using the following primers and PCR cycles. Cycle conditions were as follows: 94°C for 2 min; followed by 33 cycles of 94°C denaturation for 10 s, 58°C annealing for 15 s, and 72°C elongation for 30 s; with a final incubation at 72°C for 2 min in a PCR Thermal Cycler (BIOMETRA). PCR products were separated by electrophoresis and stained with ethidium bromide. The primers for PCR are as follows; EZH2 exon9 F, 5′-AAGCGGAAGAACACAGAAAC-3′, EZH2 exon10 R, 5′-CAGAGGAGCTCGAAGTTTCA-3′, For quantitation analysis of alternative splicing products, the signals were measured by ImageJ software [U.S. National Institutes of Health, Bethesda (Schneider et al., 2012)].

## Author Contributions

SM and NK started this project, conceived and designed the experiments. SM, AH, YS, AK, and NK performed the experiments including RNA sequencing. SI and NK analyzed RNA sequencing results. SM, SI, AH, YS, AK, SO, FH, S-IT, and NK analyzed the data. SM, SI, AH, YS, AK, KS, FH, S-IT, and NK contributed reagents, materials, and analysis tools. SM and NK wrote the manuscript. NK took the primary responsibility for the final content. SM, SI, AH, YS, AK, SO, KS, FH, S-IT, and NK read and approved the final manuscript.

## Conflict of Interest Statement

The authors declare that the research was conducted in the absence of any commercial or financial relationships that could be construed as a potential conflict of interest.
